# Scrub typhus‐associated movement and gait disorders: A systematic review with principal component analysis and in silico mechanistic modelling

**DOI:** 10.1111/tmi.14114

**Published:** 2025-04-21

**Authors:** Ritwick Mondal, Shramana Deb, Mrinmay Dhauria, Purbita Sen, Vramanti Sarkar, Shramana Sarkar, Dipanjan Chowdhury, Durjoy Lahiri, Jayanta Roy, Julián Benito‐León

**Affiliations:** ^1^ Department of Neurology Manipal Groups of Hospitals Kolkata India; ^2^ Department of Biotechnology Regional Centre for Biotechnology Faridabad India; ^3^ SN Pradhan Centre for Neuroscience University of Calcutta Kolkata India; ^4^ Department of Internal Medicine IPGMER and SSKM Hospital Kolkata India; ^5^ Division of Neurology, Department of Medicine Queen's University Kingston Ontario Canada; ^6^ Department of Neurology 12 de Octubre University Hospital Madrid Spain; ^7^ Group of Neurodegenerative Diseases Hospital Universitario 12 de Octubre Research Institute (imas12) Madrid Spain; ^8^ Centro de Investigación Biomédica en Red Sobre Enfermedades Neurodegenerativas (CIBERNED) Madrid Spain; ^9^ Department of Medicine Complutense University Madrid Spain

**Keywords:** cerebellar ataxia, gait abnormalities, immune‐mediated dysfunction, in‐silico analysis, molecular mimicry, movement disorders, neuroinflammation, opsoclonus‐myoclonus syndrome, parkinsonism, principal component analysis, protein–protein interactions, scrub typhus, sensory ataxia

## Abstract

**Background:**

*Orientia tsutsugamushi*, the causative agent of scrub typhus, is increasingly recognised for its neurological complications. Among these, movement and gait disorders are poorly understood. We systematically examined their clinical spectrum and explored underlying mechanisms through in‐silico protein–protein interaction modelling.

**Methods:**

A systematic review was conducted following PRISMA guidelines, including studies published up to 5 November 2024. Principal component analysis was used to identify clinical patterns among neurological features. In‐silico protein–protein interaction modelling was used to examine potential cross‐reactivity between *Orientia tsutsugamushi* proteins and human targets proteins.

**Results:**

Among 76 cases, 50 presented with either isolated or combined movement disorders, most commonly opsoclonus (64.0%, 32/50), with the opsoclonus‐myoclonus combination predominating (59.4%, 19/32). Other hyperkinetic features included tremor (4.0%, 2/50) and distinct forms of myoclonus (without opsoclonus) (8.0, 4/50%). Parkinsonism was present in 26.0% (13/50) of cases. Gait disorders, excluding parkinsonian gait and instability due to myoclonus, were well‐characterised in 27 patients, one of whom had concomitant opsoclonus and cerebellar ataxia. Ataxic gait was the predominant pattern, observed in 96.3% (26/27), primarily cerebellar in origin. Principal component analysis revealed five principal components reflecting distinct clinical clusters: cerebellar dysfunction, tremor and parkinsonism, sensory ataxia and spinal involvement, myoclonus (diaphragmatic/action/segmental), and prolonged recovery and cranial nerve involvement. In‐silico analyses revealed high‐confidence interactions between bacterial epitopes and host proteins, including fibronectin‐1 and Golgi‐associated molecules, suggesting mechanisms of immune‐mediated injury and neuroinflammation.

**Conclusions:**

Scrub typhus may lead to a range of movement and gait disorders through neuroimmune mechanisms and molecular mimicry. Principal component analysis offered a data‐driven framework to classify these manifestations, highlighting clinically relevant patterns. Early recognition and targeted treatment are critical to improving outcomes. Future studies should validate the molecular targets identified and evaluate immunomodulatory strategies for therapeutic intervention.

## INTRODUCTION

Movement and gait disorders often co‐occur due to shared or interconnected neural pathways, and evaluating them in tandem provides a more comprehensive understanding of motor dysfunction. These syndromes are increasingly reported as complications of infectious diseases, manifesting during the acute phase or as para‐ or post‐infectious phenomena [1, 2, 3, 4]. Their often delayed onset contributes significantly to patient morbidity and underscores the need for early recognition and targeted therapeutic intervention.

Scrub typhus, caused by *Orientia tsutsugamushi*, is a re‐emerging mite‐borne infection endemic to Southeast Asia and the Western Pacific [5]. Although often self‐limiting, delayed diagnosis may result in severe systemic involvement [6]. The rising prevalence of scrub typhus—fueled by ecological shifts and an increase in arthropod‐borne infections—has elevated its public health importance, with serious neurological complications, including encephalitis, meningitis, meningoencephalitis, and myelitis, now recognized as key contributors to mortality beyond classical systemic involvement [7, 8].

Among neurological complications, movement and gait disorders are being increasingly recognised but remain underdiagnosed due to overlapping features and limited mechanistic understanding. Accurate identification of these manifestations is critical for guiding timely treatment and preventing long‐term disability.

This systematic review, incorporating principal component analysis and in‐silico modelling, aims to characterise the clinical patterns of movement and gait disorders associated with scrub typhus, explore potential pathogenic mechanisms, and provide actionable insights for clinicians, neuroscientists, and public health professionals.

## METHODS

### Design

The systematic review followed PRISMA guidelines (CRD42024592204) and included studies focusing on confirmed movement or gait disorders associated with scrub typhus while excluding cases with prior cortical, subcortical, or spinal cord disease.

### Search strategy

A comprehensive search of PubMed, EMBASE, Cochrane Library, and Web of Science was conducted through 5 November 2024, with terms related to scrub typhus and movement or gait disorders. Relevant Medical Subject Headings (MeSH) and keywords included ‘Scrub typhus’, ‘movement disorders’, ‘hypokinesia’, ‘hyperkinesia’, ‘cerebellar disorders’, ‘ataxia’, ‘myoclonus’, ‘gait abnormality’, ‘parkinsonism’, ‘myelitis’, and ‘dyskinesias’. Manual searches of reference lists, journal websites, and preprint servers (e.g., medRxiv, bioRxiv) supplemented the search, covering studies from 1991 onward.

### Screening process

Three reviewers (DC, VS, and SS) conducted the initial screening process, with all literature imported into Rayyan software for duplicate removal. Two reviewers (PS and DL) then screened titles and abstracts for relevant cases, and three others (RM, SD, and MD) applied eligibility criteria to the full texts. Discrepancies were resolved by consensus with input from JR and JBL.

### Study selection criteria

We included peer‐reviewed and preprint cohort studies, case–control studies, case series, and case reports involving scrub typhus‐positive patients with documented hyperkinetic or hypokinetic movement disorders or gait abnormalities. Only studies published in English were considered. Exclusion criteria included studies without microbiologically confirmed scrub typhus diagnosis, non‐English publications, reviews, opinion pieces, and reports lacking specific data on movement or gait disorders.

### Data extraction

Data extraction was performed using piloted forms to ensure consistency, capturing details on study design, population characteristics, outcomes, and study limitations.

Although a formal meta‐analysis was not feasible due to the descriptive nature of the data, exploratory analyses—including descriptive synthesis, principal component analysis, and correlation modelling—were performed by RM, SD, and MD. Tables were constructed to systematically summarize the key findings.

### Quality assessment

The Newcastle‐Ottawa scale evaluated study quality, focusing on selection procedures, comparability, and outcomes.

### Statistical analysis

All statistical procedures were performed using GraphPad Prism (v5.0) and JASP (Jeffrey's Amazing Statistics Program, version 0.17.2) [9].

*Pearson's correlation test*: Correlations between variables were assessed using Pearson's correlation test in JASP software. Adjustments were made to report significance, flag significant correlations, and generated a heatmap displaying the correlation coefficients (r) and significance levels (indicated by an asterisk). The *X* and *Y* axes represented the correlated variables, with each box in the heatmap indicating the direction and strength of the correlation.

*Principal component analysis*: Recognised as a cornerstone statistical method, principal component analysis enables the reduction of high‐dimensional clinical data into key components that account for the greatest variance across the dataset [10, 11]. Using principal component analysis, researchers can identify patterns and relationships in complex data, providing insights that might otherwise remain obscured. In this study, we utilised JASP to analyse high‐dimensional clinical data, aiming to identify movement and gait disorders and their associated neurological manifestations in cases of scrub typhus. This approach enabled us to extract the most informative features, facilitating a clearer understanding of the clinical spectrum associated with this condition.

*Principal component analysis input options*: Components were selected through parallel analysis, with factors retained if their eigenvalues exceeded the average random eigenvalue. Components with eigenvalues greater than one were automatically selected. An oblique rotation method (Promax) was applied to facilitate correlations between components, thereby enhancing interpretability. Principal component analysis was based on the correlation matrix by default.

*Principal component analysis output options*: Loadings greater than 0.4 were displayed, with component sizes ordered by loading. Path diagrams and scree plots were generated to visualise results from parallel analysis.

In principal component analysis, interpreting both positive and negative loadings offers valuable insights into the relationships between variables. A negative loading indicates that as the variable value increases, the corresponding principal component score tends to decrease, and vice versa. This inverse dynamic is particularly informative, as it reveals potential opposing trends among variables. For example, when one variable shows a positive loading and another a negative loading on the same component, it suggests a meaningful inverse relationship between them. Interpreting these loadings enriched our analysis by revealing latent structures and underlying patterns within the data.

### In‐silico study for cross‐antigenic reactivity

In‐silico analyses are powerful computational approaches used to investigate complex biological data. They help identify patterns, predict disease mechanisms, and explore molecular interactions in ways that might not be possible with traditional methods [12, 13].

We systematically searched the PubMed, EMBASE, Cochrane Library, and Web of Science databases to identify *Orientia tsutsugamushi* surface epitope proteins conserved across serotype variations and their significant interactions with target host proteins. Following curation, we employed the Search Tool for the Retrieval of Interacting Genes/Proteins (STRING, version 12.0) (https://string-db.org/) to generate predictive protein–protein interaction (PPI) network models for each epitope within host cellular context.

Using gene symbols retrieved from the National Center for Biotechnology Information (NCBI), query proteins were uploaded to STRING for analysis. The resulting networks depicted both functional and physical interactions, supported by multiple evidence sources, including text mining, co‐expression patterns, curated databases, gene neighbourhood, fusion events, co‐occurrence, and protein homology. A high‐confidence interaction score threshold (≥0.700) was applied to ensure robustness. To further delineate interaction patterns, we performed *k*‐means clustering (number of clusters: 2), allowing the identification of distinct clusters among the target host proteins.

The brain‐tissue‐specific expression profiles of the STRING‐predicted target host proteins were analysed using data from the GTEx Portal (www.gtexportal.org).

The use of these two analytical methods is well supported in the literature for investigating the complex interplay between infectious agents and the host’s neurological system. Collectively, they offer a robust framework for elucidating the intricate mechanisms underlying movement and gait disorders associated with scrub typhus.

## RESULTS

**FIGURE 1:**
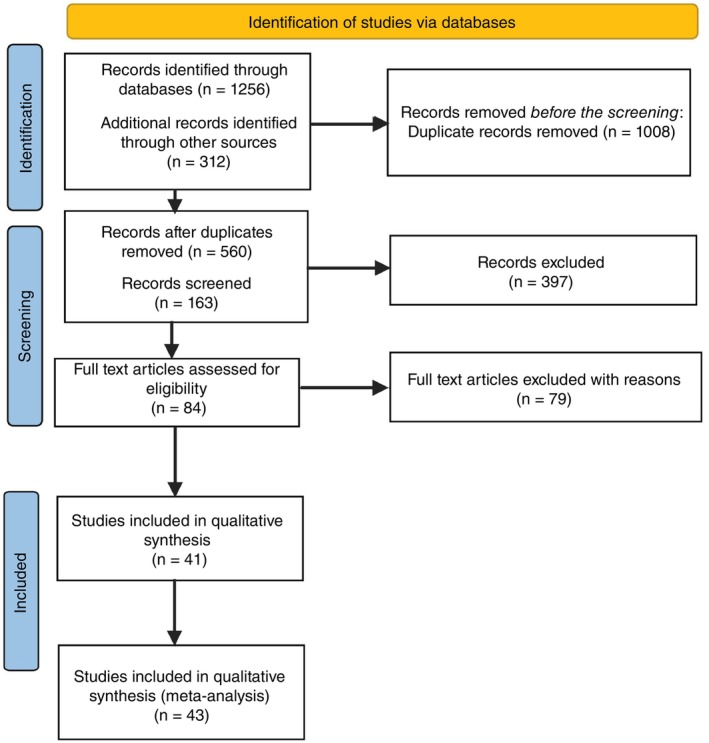
PRISMA flow chart depicting study selection criteria.

We initially identified 1256 articles from databases and 312 from preprint servers. After removing duplicates, 560 unique articles remained. Following title and abstract screening, 163 articles were selected for full‐text review. Applying the inclusion and exclusion criteria led to the elimination of 79 articles, including those based on study type (e.g., review papers, correspondence, viewpoints, or commentaries). Ultimately, 84 articles were selected for inclusion, with 43 articles chosen for detailed analysis [14, 15, 16, 17, 18, 19, 20, 21, 22, 23, 24, 25, 26, 27, 28, 29, 30, 31, 32, 33, 34, 35, 36, 37, 38, 39, 40, 41, 42, 43, 44, 45, 46, 47, 48, 49, 50, 51, 52, 53, 54, 55, 56, 57], while the remaining 41 were synthesised narratively (Figure 1).

We analysed 76 cases of movement and gait disorders associated with scrub typhus extracted from 43 articles published between 1991 and 2024. Demographic data were available for the majority of cases, with sex reported in 81.6% (62/76) of cases, indicating a male predominance (23 women vs. 39 men). Age was documented in 80.3% (61/76) of cases, ranging from 3 to 73 years (mean 32.2 years), with most patients being adults aged 18–60 years (68.8%, 42/61), followed by paediatric cases under 18 years (18.0%, 11/61) and patients over 60 years (13.1%, 8/61) (Table 1).

**TABLE 1:** Scrub typhus cases with movement and gait disorders.

Author (year)	Cases	Age/sex	Comorbidities	Systemic symptoms	Neurological symptoms	CSF analysis	Serum analysis	Diagnostic test	Movement or gait disorder	Latency	Treatment	Outcome
Silpapojakul et al. [14] (1991)	1	15 Y/M	Renal insufficiency, jaundice, and uveitis	Fever, headache, and vomiting	Generalised tonic–clonic seizures, slurred speech, and nystagmus	Glucose: 66 mg/dL and protein: 107 mg/dL	Not significant	Scrub typhus IgM (ELISA)	Dysdiadokokinesis, intentional tremor, and ataxic gait	Not reported	Not reported	Not reported
Kim et al. [15] (2008)	1	64 Y/M	Hearing impairment	Fever, myalgia, dizziness, sinus tachycardia, and orthostatic hypotension	Dysarthria, altered sensorium, neck stiffness, and reduced sensation in all limbs	Protein: 151 mg/dL and glucose: 46 mg/dL	Thrombocytopenia, leukocytosis, and elevated liver enzymes	Serum IFA	Demyelinating sensorimotor polyneuropathy with wide‐based ataxic gait	14 days	Doxycycline 200 mg × 14 days	Neurological improvement after 3 months
Yum et al. [16] (2011)	1	30 Y/M	Not reported	Fever, weakness, headache, diplopia, malaise, and eschar	Left abducens nerve palsy, nystagmus, diplopia, and decreased motor strength	Lymphocytic pleocytosis, glucose: 80 mg/dL, and protein: 120 mg/dL	Elevated transaminases and increased C‐reactive protein	Scrub typhus IgM (ELISA)	Meningoencephalitis with ataxic gait	3 days	Doxycycline 100 mg BID × 20 days	Complete recovery
Chiou et al. [17] (2013)	1	55 Y/M	Not significant	Eschar on trunk and limbs, fever, headache, and malaise	Not significant	Not reported	Not significant	IFA positive for IgM against *Orientia tsutsugamushi*	Transient parkinsonism with bilateral upper limb tremor, wide‐based gait, and myoclonus	2 days	Doxycycline 100 mg BID, amantadine 100 mg BID, and clonazepam 1 mg TDS	Discharged with mild postural tremor at 1 month
Karanth et al. [18] (2013)	1	24 Y/M	Not reported	Drowsiness, fever, headache, and eschar on right thigh	Ataxic speech, horizontal gaze, and nystagmus	Lymphocytic pleocytosis and protein: 60 mg/dL	Neutrophilia, thrombocytopenia, elevated transaminases, and CK	Scrub typhus IgM (IFA)	Bilateral cerebellar dysfunction with dysdiadokokinesia and truncal ataxia	12 days	Ceftriaxone and doxycycline × 14 days	No residual deficit
Bhat et al. [19] (2015)	1	6 Y/F	Not reported	Fever and enlarged cervical lymph nodes	Scanning speech, generalised tonic–clonic seizures	Normal	Elevated erythrocyte sedimentation rate and C‐reactive protein	Weil‐Felix test positive (OXK titre 1:320)	Ataxic gait with cerebellar signs	Not reported	Not reported	Not reported
Kim et al. [20] (2015)	1	73 Y/M	Arterial hypertension	Not significant	Not significant	Not reported	Multiple abnormalities	PHA test positive, scrub typhus IgM+ (1:128)	Transient parkinsonism with bilateral bradykinesia, resting and postural tremor, and rigidity	5 days before the presentation	Doxycycline 200 mg × 5 days	Significant improvement at 2 weeks
Koti et al. [21] (2015)	1	26 Y/M	Not significant	Fever, breathlessness	Myoclonus, opsoclonus, and hyperreflexia with increased muscle tone	Normal	Leukocytosis with lymphocytosis and elevated liver enzymes	Scrub typhus IgM+	Opsoclonus‐myoclonus, head titubation, and saccadomania	6 days	Doxycycline	Asymptomatic upon discharge
Premaratna et al. [22] (2015)	1	62 Y/M	Not significant	Fever, rigour, eschar, bibasal crepitation	Mask‐like face, right‐sided increased muscle tone, and normal reflexes	Not reported	WBC 13,400, lymphocyte predominant	IFA positive for IgM and IgG	Parkinsonism with right‐sided resting tremor and stiffness	5 days	Doxycycline and azithromycin	Fully improved within 2 weeks
Bhoil et al. [23] (2016)	1	21Y/M	Not significant	Fever, rash	Slurred speech	Not significant	Not reported	Scrub typhus IgM (Weil‐Felix, ELISA)	Acute cerebellitis with severe ataxia	Not reported	Not reported	Not reported
Mahajan et al. [24] (2017)	1	22 Y/F	Not reported	Fever, headache, body aches, vomiting, jaundice, and conjunctival suffusion	Scanning speech, square wave jerks, hypotonia	Protein: 90 mg% and 15 WBC (all lymphocytes)	Multiple abnormalities	Serum scrub typhus IgM (ELISA)	Cerebellar dysfunction with impaired coordination, truncal and gait ataxia	9 days	Doxycycline 100 mg BID × 14 days and dexamethasone 4 mg TDS × 10 days	Improved at 4 weeks
Rana et al. [25] (2017)	5	Not reported	Not reported	Fever and altered sensorium	Not reported	Not reported	Raised CPK in the neuroleptic malignant syndrome case	Scrub typhus IgM (ELISA)	4 cases: Cerebellitis with ataxia and one with neuroleptic malignant syndrome	Not reported	Not reported	Not reported
Ghosh et al. [26] (2017)	1	3Y/F	Not significant	Fever, irritability, lymphadenopathy and hepatosplenomegaly	Photophobia and nystagmus	Normal glucose and protein; lymphocytic pleocytosis	Thrombocytopenia, elevated transaminases, and raised C‐reactive protein	Serum scrub typhus IgM (ELISA)	Acute cerebellitis with truncal and peripheral ataxia	5 days	IV ceftriaxone and oral doxycycline × 10 days	Complete recovery at 1 week
Sahu et al. [27] (2017)	1	60 Y/M	Acute respiratory distress syndrome	Fever, chills, and jaundice	Drowsy (Glasgow Coma Scale 12/15) and saccadic eye movements	Not significant	Multiple abnormalities	IFA and IgM positive (1:128)	Opsoclonus and multiaxial involuntary saccades	Not reported	Doxycycline, azithromycin, and steroids	Complete resolution
Rajesekar and Ameen [28] (2017)	1	M (age not reported)	Not reported	High‐grade fever, chills, vomiting, episcleritis, lymphadenopathy, and eschar	Emotional lability, left upper motor neuron facial palsy, and exaggerated reflexes	Protein: 146.3 mg/dL, glucose: 45 mg/dL, and total count: 50 cells	Leukocytosis with differential changes, elevated transaminases	Scrub typhus IgM (ELISA)	Acute disseminated encephalomyelitis with opsoclonus, bilateral cerebellar signs, cogwheel rigidity, and gait ataxia	Not reported	Doxycycline 100 mg BID	Gradual improvement
Didel et al. [29] (2017)	1	9 Y/M	Not significant	Fever, headache, vomiting, conjunctival suffusion, and icterus	Nystagmus and left‐sided truncal ataxia	Glucose: 94 mg/dL, and protein: 60 mg/dL	Lymphocytic leukocytosis and elevated liver enzymes	IgM ELISA and PCR‐positive	Acute cerebellitis with left‐sided truncal ataxia	Not reported	Oral doxycycline	Symptoms resolved in 1 week
Nandi et al. [30] (2018)	1	3 Y/M	Not significant	High‐grade intermittent fever and irritability	Saccades	Pleocytosis, glucose: 92 mg/dL, and protein: 32 mg/dL	Not significant	IgM ELISA positive	Opsoclonus with generalised myoclonus affecting all limbs	12 days	Doxycycline × 10 days	Complete recovery at 3 months
Kasinathan et al. [31] (2019)	1	9 Y/M	Not reported	Subacute fever, hepatosplenomegaly, and scrotal eschar	Conjugate horizontal saccadic oscillations, right convergent squint, and ocular flutter	Lymphocytic pleocytosis (70 cells) and protein: 105 mg/dL	Not reported	IgM ELISA positive	Acute cerebellitis with cerebellar ataxia	Not reported	IV doxycycline and dexamethasone × 5 days	Complete recovery in 5 days
Kamalasanan and Kiran [32]. (2019)	1	70 Y/F	Acute kidney injury	Fever, chills, myalgia, breathlessness, oliguria, tachycardia, hypoxia	Recent onset memory impairment	Protein: 15 mg/dL and glucose: 69 mg/dL	Increased erythrocyte sedimentation rate	Serum scrub typhus IgM (ELISA)	Parkinsonism and myoclonus	2 days	Doxycycline, azithromycin, and clonazepam	Parkinsonism resolved at 7 days, persistent memory impairment
Paul and Thakur et al. [33] (2019)	1	40 Y/M	Not reported	Fever, headache, vomiting, and drowsiness	Neck rigidity	Pleocytosis, glucose: 56 mg/dL and protein: 121 mg/dL	Elevated transaminases and leukocytosis	IgM ELISA positive	Encephalitis with opsoclonus‐myoclonus	2 days	Doxycycline	Not reported
Neela et al. [34] (2024)	1	34 Y/M	Not significant	Fever and headache	Progressively blurred vision	Mildly elevated protein (71 mg/dL), normal glucose, and mild pleocytosis (11 cells/mm³)	Normal	Scrub typhus IgM ELISA	lsolated opsoclonus	10 days	Doxycycline and azithromycin	Complete recovery
Sardana et al. [35] (2019)	1	10 Y/M	Not significant	Fever and headache	Vertigo, diplopia, speech difficulty, bilateral sixth cranial nerve palsy, and papilledema	Increased protein (91 mg/dL) and glucose: 45 mg/dL	Increased total leukocyte count	Scrub typhus IgM ELISA	Acute disseminated encephalomyelitis with dysdiadochokinesia and ataxic gait	Not reported	Azithromycin, doxycycline, and steroids	Partial recovery in 1 week
Ralph et al. [36] (2019)	18	38 ± 16.8 Y/9M and 9F	Diabetes in 2 cases Hypertension in 1 case, and chronic lung disease in 1 case	Headache in 13 cases, nausea/vomiting in 12 cases, and eschar in 8 cases	Seizures and altered sensorium, meningitis (*N* = 3)	Elevated protein in 14 cases; pleocytosis in 14 cases; and elevated glucose in 11 cases	Multiple abnormalities	IgM ELISA; PCR confirmation	Opsoclonus, myoclonus, and parkinsonism features	7 days (median)	Doxycycline, azithromycin, and other supportive care	Most resolved at 3 months
Gupta et al. [37] (2020)	1	26 Y/F	Not reported	Fever with chills, rigour, headache, vomiting	Hypotonia	Glucose: 50 mg/dL and protein: 35 mg/dL	Thrombocytopenia, elevated transaminases	Scrub typhus IgM ELISA (Serum)	Dysdiadokokinesia, pendular and horizontal gaze nystagmus, cerebellar dysfunction associated with broad‐based gait and truncal ataxia	7 days	Antipyretics and doxycycline	Complete recovery except nystagmus
Kaiser et al. [38] (2020)	1	7 Y/F	Not significant	High‐grade fever, headache, projectile vomiting, and vision impairment	Latent right eye squint, nuchal rigidity	CSF WBC: 102/cm3 (92% lymphocytes), Protein: 102 mg/dL	Microcytic anaemia, leukocytosis	CSF and blood scrub IgM positive	Cerebellar ataxia with unsteady gait, tendency to fall	11 days	Multiple treatments, including doxycycline	Complete recovery
Ryu et al. [39] (2020)	1	66 Y/M	Not reported	Myalgia, fever, headache, abdominal eschar	Paresthesia, urinary retention, loss of tendon reflex	Glucose: 59 mg/dL, Protein: 67 mg/dL	WNL	CSF IgG (1:110), Serum IFA (1:2560)	Gait and balance impairment due to acute transverse myelitis	5 days	Methylprednisolone, doxycycline	Normal gait at 1 year
Soundararajan et al. [40] (2020)	1	50 Y/M	Not significant	Fever, cough, breathlessness, insomnia, disorientation, hepatomegaly	Hypophonia, slurred speech	Protein: 85 mg/dL	Elevated transaminases, thrombocytopenia, neutrophilia	Serum Scrub Typhus IgM (ELISA)	Resting tremors, shuffling gait	15 days	Fluid therapy, ceftriaxone, doxycycline	Complete recovery in 10 days
Venkatesh et al. [41] (2021)	1	18 Y/M	Not reported	Fever, petechial rash, conjunctival suffusion, vomiting	Scanning speech, dysarthria, nystagmus, sensory loss	Not reported	Thrombocytopenia, decreased albumin, elevated liver enzymes	IgM positive (rapid test)	Broad‐based gait, left‐sided cerebellar signs	7 days	Ceftriaxone and doxycycline	No recurrence at 4 months
Garg et al. [42] (2021)	1	23 Y/F	Not significant	Fever, eschar (left sub‐mammary), bibasal crepitation	Not significant	Normal	Multiple abnormalities, including thrombocytopenia, elevated BUN	Scrub typhus IgM (ELISA)	Opsoclonus‐myoclonus syndrome	2 days	IV azithromycin × 10 days	Resolution over 2 weeks
Ninama [43] (2021)	1	15 Y/M	Not significant	Fever, drowsiness, eschar on right thigh	Slurred speech	Aseptic meningitis pattern: 32 cells (70% lymphocytic), normal glucose/protein	Multiple abnormalities, including thrombocytopenia	Serum scrub typhus IgM (ELISA)	Cerebellar ataxia with truncal involvement	3 days	Oral doxycycline, IV fluids, empiric antibiotics	Dramatic improvement at 48 h, complete recovery at 8 days
Raghunathan et al. [44] (2022)	1	7 Y/M	Not reported	High‐grade fever, drowsiness	Not significant	Elevated Protein (60 mg/dL), Glucose (70 mg/dL)	CPK‐ 28 U/L	Serum scrub typhus IgM antibodies by ELISA	Guillain‐Barré syndrome with sensory ataxia, quadriparesis, and areflexia	3 Days	Azithromycin	After 1‐month follow‐up: Complete neurological recovery
Ghosh et al. [45] (2022)	1	35 Y/M	Not significant	Febrile and tachycardia	Right side upper motor neuron facial palsy, brisk deep tendon reflexes, and Babinsky. Decreased pain and temperature sensation on the left half of his trunk and limbs. Right‐sided partial/incomplete Horner's syndrome	Mildly elevated protein (60 mg/dL) low glucose (35 mg/dL) lymphocytic pleocytosis (15 cells; all lymphocytes)	Neutrophilic leukocytosis (81% neutrophils), Elevated erythrocyte sedimentation rate (58 mm in the first hour), and thrombocytopenia (96,000/uL).	Serum: scrub typhus IgM antibodies with ELISA CSF: Scrub typhus positive by PCR	Right sided spastic hemiperesis (mRC 4/5) Truncal and gait ataxia (unsteadiness) with a tendency to fall on the right (Associated with Opalski's syndrome)	Concomitant onset with fever onset	Doxycycline	At discharge: only mild impairment of sensation over the left half of his body and facial pain over the right half of his face, for which he was prescribed pregabalin at bedtime. Follow‐up 3 months: persistence of mild sensory abnormalities.
	1	62 Y/F	Not significant	High‐grade fever, chills, myalgia, headache, anorexia, and mild jaundice	Hypophonic speech, masked facies	Raised protein (68 mg/dL) with lymphocytic pleocytosis (21 cells, all lymphocytes) with low glucose levels (23 mg/dL)	Anaemia, leukopenia with relative lymphocytosis, thrombocytopenia, and raised erythrocyte sedimentation rate	Serology for scrub typhus (both IgM‐ELISA and Weil‐Felix) was positive	New‐onset symmetrical resting tremor, axial and appendicular rigidity, and severe slowness of all body movements (bradykinesia to akinesia)	Concomitant onset with fever onset	Oral doxycycline, pramipexole levodopa‐carbidopa, and sertraline	Three months of follow‐up: mild Parkinsonian features; follow‐up after 1 year revealed no further relapse or neurological deterioration.
	1	28 Y/F	Not significant	Fever, tachycardia	Papilledema, neck rigidity, positive Brudzinski's sign, scanning/ataxic speech	Decreased glucose (30 mg/dL), high protein (80 mg/dL) and lymphocytic pleocytosis (45 cells, all lymphocytes)	Neutrophilic leukocytosis, thrombocytopenia, raised erythrocyte sedimentation rate, and transaminitis	Serology for scrub typhus (both IgM‐ELISA and Weil‐Felix) was positive	Intention tremor, cerebellar nystagmus, frequent square wave jerks, hypotonia, impaired coordination (dysdiadokinesia), pendular knee jerks, truncal and gait ataxia	10 days	Doxycycline and dexamethasone	On discharge after 4 weeks: Ability to walk without support with subtle cerebellar dysfunction. Follow‐up visit: After 8 weeks, there were improved signs of cerebellar dysfunction.
	1	40 Y/F	Not significant	Fever and Headache	Not significant	Raised protein (88 mg/dL) and low glucose (29 mg/dL).	Anaemia, lymphocytic leukocytosis, mild thrombocytopenia, raised erythrocyte sedimentation rate, mild asymptomatic hyponatremia, raised transaminase, and low albumin	Scrub Typhus IgM (ELISA followed by immunochromatographic card test)	Isolated opsoclonus	10 days	Doxycycline	Sixth month of follow‐up: No sign/symptoms of relapse/recurrence of similar episodes
Ramkumarsingh Tomar et al. [46] (2022)	1	28Y/F	Not Reported	Fever	Chaotic eye movement	Cells: 45/mL (100% lymphocytes), Increased protein: 99 mg/dL, decreased glucose: 28.3 mg/dL	Leucocytosis (16,109 cells/L), thrombocytopenia, and elevated serum transaminase levels	Scrub typhus IgM (ELISA)	Opsoclonus‐ myoclonus with imbalance while walking and recurrent falls.	2 days	Doxycycline clonazepam	At one‐year follow‐up: no relapse of opsoclonus‐myoclonus syndrome
Ghosh et al. [47] (2022)	1	45 Y/M	Not significant	Fever, vomiting, dysphagia, headache	Decreased hearing ability, generalised tonic–clonic seizures, neck rigidity, CN‐VII, IX, X involvement	Protein: 160 mg/dL, glucose: 30 mg/dL and lymphocytic pleocytosis	Thrombocytopenia and anaemia	PCR confirmation (serum and CSF)	Diaphragmatic myoclonus, action myoclonus, myoclonus involving shoulder girdle	3 days	Doxycycline and azithromycin	Most resolved within 8 days
Cho et al. [48] (2023)	1	64 Y/M	Not significant	Fever	Difficulty in defecation, reduced sensation below T4, reduced sensation in the groin and inguinal area, allodynia in both legs, impaired proprioception with a positive Romberg sign, and absent deep tendon reflexes	Not significant	Not significant	Serum scrub typhus IgG antibodies by ELISA	Myelopathy concomitant with severe sensory dominant polyneuropathy associated with high‐stepping gait	21 days	IV steroid therapy	Substantial improvement
Dalamapati et al. [49] (2023)	1	35 Y/M	History of varicella‐zoster virus encephalitis	Fever, gastrointestinal symptoms and rash	Cerebellar dysarthria	Lymphocytic pleocytosis and protein: 64.5 mg/dL	Multiple abnormalities	Scrub typhus IgM (ELISA)	Cerebellar dysfunction with multiple manifestations	5 days	Methylprednisolone, doxycycline	Significant improvement at 30 days
Damodar et al. [50] (2023)	8	Not reported	Not reported	Fever, altered sensorium	Not reported	Not reported	Not reported	IgM ELISA positive	Opsoclonus and myoclonus, choreoathetosis movements and hemiballismus, abnormal perioral movements, lip smacking, teeth grinding, and rapid eye blinking occurred in 1 patient each; tremors occurred in 2 patients	Not reported	Not reported	Not reported
Nenjnktpaln [51] (2023)	1	64 Y/M	Not reported	Fever	Not significant	Normal protein/glucose, pleocytosis	Not reported	IgM ELISA positive	Generalised opsoclonus‐myoclonus	7 days	Multiple treatments, including steroids	Reduced symptoms at 2 weeks
Biswas et al. [52] (2024)	1	23 Y/F	Not significant	Altered sensorium, high‐grade fever	Multiple neurological signs, including papilledema	Multiple abnormalities, including elevated protein	Not significant	Serum and CSF scrub typhus IgM positive	Meningoencephalitis with cortical multifocal myoclonus	1 day	Complex regimen including multiple anticonvulsants	Complete resolution at 3 months
Singh et al. [53] (2024)	1	18 Y/F	Not reported	High‐grade fever, headache, vomiting	Generalised tonic–clonic seizures and nuchal rigidity	Elevated protein/glucose, pleocytosis	Multiple abnormalities	IgM ELISA positive	Generalised opsoclonus‐myoclonus	10 days	Multiple treatments, including antiepileptics	Complete recovery in 11 days
Rehani et al. [54] (2024)	1	Not reported	Significant comorbidities	Fever	Multiple neurological signs	Normal	Not significant	Blood scrub typhus IgM positive	Bilateral symmetrical parkinsonism	7 days	Doxycycline and rifampicin	Bed bound status at discharge
Puppala et al. [55] (2024)	2	14 Y/M, 30 Y/M	Not significant	High‐grade fever, chills, rigours	Variable including generalised tonic–clonic seizures	Variable findings	Not significant	Blood scrub typhus IgM positive	Case 1: opsoclonus‐myoclonus; case 2: generalized myoclonus and rigidity (Movement Disorder Society‐Unified Parkinson's disease Rating Scale Grade II)	3–9 days	Doxycycline × 14 days	Complete resolution in both cases
Yarlagadda et al. [56] (2024)	1	22 Y/M	Dengue co‐infection	Fever, headache, and vomiting	Grade‐II papilledema	Elevated intracranial pressure and pleocytosis	Not reported	IgM ELISA positive	Encephalitis with isolated opsoclonus‐myoclonus	5 days	Multiple antibiotics	Complete recovery within 2 weeks
Dave et al. [57] (2024)	1	21 Y/F	Not reported	High‐grade fever, nausea/vomiting	Multiple neurological signs	Protein: 184 mg/dL and Cells: 178/mm^3^ (90% lymphocytes).	Not reported	Scrub typhus IgM (ELISA)	Bilateral cerebellar ataxia with multiple manifestations	4 days	Multiple antibiotics, including doxycycline	Discharged on treatment

Abbreviations: ALT, alanine aminotransferase; AST, aspartate aminotransferase; BUN, blood urea nitrogen; CSF, cerebrospinal fluid; CK, creatine kinase; ELISA, enzyme‐linked immunosorbent assay; F, female; IgG, immunoglobulin; IgM, immunoglobulin; IFA, indirect immunofluorescence assay; IV, intravenous; M, male; mRC, modified Rankin Scale; PCR, polymerase chain reaction; TLC, total leukocyte count; WBC, white blood cell count; Y, years.

In general, clinical characteristics were well documented across the series. Among 48 (63.1%) cases with reported comorbidity status, nearly half (45.8%, 22/48) had no significant pre‐existing conditions. Clinical findings were documented in all cases. The most frequent clinical signs included fever (72.4%, 55/76), vomiting (31.6%, 24/76), eschar (22.4%, 17/76), and altered sensorium (28.9%, 22/76). The predominant reported symptom was headache (39.5%, 30/76) (Table 1).

Among 76 cases, 50 presented with either isolated or combined movement disorders, with opsoclonus (64.0%, 32/50) being the most common. Combined opsoclonus‐myoclonus predominated (59.4%, 19/32), while opsoclonus without myoclonus was less frequent (40.6%, 13/32). Other hyperkinetic disorders included tremors in 4.0% (2/50), distinct forms of myoclonus (without opsoclonus) in 8.0% (4/50), and rare occurrences of choreoathetosis and hemiballismus in 2.0% (1/50) of cases. Parkinsonism was relatively frequent, observed in 26.0% (13/50) of cases. Additionally, neuroleptic malignant syndrome was reported in 2.0% (1/50) of cases, presenting with severe muscle rigidity (Table 1).

Gait disorders, excluding parkinsonian gait and instability due to myoclonus, were well‐characterised in 27 patients, one of whom had concomitant opsoclonus and cerebellar ataxia. Ataxic gait was the predominant pattern, observed in 96.3% (26/27). Among ataxic presentations, cerebellar ataxia was the most common (88.5%, 23/26), followed by sensory ataxia (11.5%, 3/26) (Table 1).

Laboratory investigations provided significant insights, with cerebrospinal (CSF) analysis performed in 77.6% (59/76) of cases. Common CSF abnormalities included elevated protein (61.0%, 36/59), pleocytosis (52.5%, 31/59), and decreased glucose (15.2%, 9/59). Serum analysis was available in 82.9% (63/76) of cases, revealing thrombocytopenia in 50.8% (32/63) and leukocytosis in 42.8% (27/63) (Table 1).

Diagnostic testing was reported in all cases, with IgM ELISA being the predominant method (88.1%, 67/76), followed by polymerase chain reaction (27.6%, 21/76) and indirect immunofluorescence assay (7.9%, 6/76). The latency period from initial symptoms to neurological manifestations was documented in 71.0% (54/76) of cases, with most patients developing symptoms within 5–10 days (64.8%, 35/54), while early‐onset (<5 days) occurred in 24.1% (13/54) and late‐onset (>10 days) in 11.1% (6/54) (Table 1).

Neuroimaging studies were reported in 53 of 76 cases (69.7%). Among these, brain magnetic resonance imaging (MRI) was the predominant imaging modality. Significant brain MRI abnormalities were documented in 17/53 cases (32.1%), while 31/53 (58.5%) showed normal or non‐significant findings. The most common abnormal findings included cerebellar involvement, seen in 12/17 cases (70.6%) with abnormal MRI, manifesting as hyperintensities on T2‐weighted/FLAIR sequences or contrast enhancement. Additional MRI abnormalities included leptomeningeal enhancement (3/17, 17.6%), brainstem involvement (3/17, 17.6%), basal ganglia lesions (3/17, 17.6%), and periventricular white matter changes (2/17, 11.8%) (Table 2).

**TABLE 2:** Neuroimaging, electrophysiological findings, and autoimmune panel in scrub typhus cases with movement and gait disorders.

Authors	No of cases	Neuroimaging findings	Electrophysiological findings (electroencephalogram, nerve conduction velocity, electromyography)	Autoimmune panel (cerebrospinal fluid/serum)
Silpapojakul et al. [14]	1	Non‐contrast computed tomography: not significant	Not reported	Not reported
Kim et al. [15]	1	Non‐contrast computed tomography and MR‐angiography showed no significant findings. However, T2‐weighted brain MRI revealed high signal intensities in the right parieto‐occipital areas and corona radiata	Not reported	Not reported
Yum et al. [16]	1	T1‐weighted MRI showed parenchymal and leptomeningeal enhancement in the brainstem, left temporal periventricular area, and right cerebellar peduncle. Fluid‐attenuated Inversion Recovery (FLAIR) MRI demonstrated hyperintensity in the brainstem, left cerebellar peduncle, and left basal ganglia. Spinal MRI findings were not significant	Not reported	Not reported
Chiou et al. [17]	1	Not reported	Not reported	Negative
Karanth et al. [18]	1	Not reported	Not reported	Not reported
Bhat et al. [19]	1	Brain MRI revealed diffuse increased signal intensity in the cerebellar cortex on T2‐weighted and FLAIR images	Not reported	Not reported
Kim et al. [20]	1	Brain MRI revealed mild atrophy in the cerebral cortex and medial temporal lobe, along with lacunar infarctions in the left basal ganglia and right cerebellum	Not reported	Not Reported
Koti et al. [21]	1	No data available	No data available	No data available
Premaratna et al. [22]	1	No data available	Normal	No data available
Bhoil et al. [23]	1	Non‐contrast computed tomography showed no significant findings. T2‐weighted FLAIR MRI revealed diffuse hyperintensity in the cerebellar cortex with restriction and post‐contrast enhancement, suggesting inflammation	Not reported	Not reported
Mahajan et al. [24]	1	T2‐weighted brain MRI demonstrated uniform enhancement of the pachymeninges accompanied by edema in both cerebellar hemispheres	Not reported	Not reported
Rana et al. [25]	Among the five cases, MRI data was available for one patient with neuroleptic malignant syndrome	Brain MRI showed no significant findings	Not reported	Not reported
Ghosh et al. [26]	1	T2‐weighted MRI revealed hyperintensity in the bilateral cerebellar cortices	Not reported	Not reported
Sahu et al. [27]	1	No data available	No data available	No data available
Rajesekar and Ameen [28]	1	Not reported	Not reported	Not reported
Didel et al. [29] (2017)	1	T2‐weighted MRI revealed hyperintensity in the inferior‐medial region of the left cerebellar hemisphere	Not reported	Not reported
Nandi et al. [30]	1	Brain MRI showed no significant findings	Not reported	Not reported
Kasinathan et al. [31]	1	Brain MRI showed no significant findings	Not reported	Not reported
Kamalasanan and Kiran [32]	1	Brain MRI showed no significant findings except for age‐related atrophic changes	Not reported	Not reported
Paul and Thakur et al. [33]	1	Brain MRI showed no significant findings	Not reported	Not reported
Neela et al. [34]	1	Normal brain computed tomography and MRI findings	Not reported	Not reported
Sardana et al. [35]	1	T2‐weighted and FLAIR MRI revealed hyperintensity in the brainstem, cerebellum, and periventricular region	Not reported	Not reported
Ralph et al. [36]	Neuroimaging data was available for three cases	T2‐weighted and FLAIR brain MRI (*n* = 3) revealed slightly increased intensity and effacement of bilateral cerebellar folia, indicating diffuse cerebellar edema. In one case (*n* = 1), T2‐weighted and FLAIR brain MRI demonstrated diffuse leptomeningeal enhancement, suggestive of meningitis	No data available	Serum and cerebrospinal fluid results were available in two patients and were negative
Gupta et al. [37]	1	Brain MRI showed no significant findings	Not reported	Not reported
Kaiser et al. [38]	1	No data available	Not reported	Not reported
Ryu et al. [39]	1	Brain MRI showed no significant findings. Spinal MRI revealed features of acute transverse myelitis, with T2‐weighted/STIR images showing intramedullary high signal intensity and spinal cord enlargement	Not reported	Not reported
Soundararajan et al. [40]	1	Non‐contrast computed tomography identified a parietal calcified granuloma	Not reported	Not reported
Venkatesh et al. [41]	1	Brain showed no significant findings	Not reported	Not reported
Garg et al. [42]	1	Brain MRI and positron emission tomography‐computed tomography showed no significant findings	Normal	Serum results were negative, while cerebrospinal fluid findings were not reported.
Ninama [43]	1	Brain showed no significant findings	Not reported	Not reported
Raghunathan et al. [44]	1	Brain and spinal MRI showed no significant findings	Not reported	Not reported
Ghosh et al. [45]	1	Brain and spinal MRI showed no significant findings	Normal	Serum findings were negative, while cerebrospinal fluid findings were not reported
	1	T2‐weighted brain MRI revealed a hyperintense lesion on the lateral right half of the medulla	Not reported	Serum and cerebrospinal fluid results were negative
	1	Brain MRI showed no significant findings	Nor reported	Serum and cerebrospinal fluid results were negative
	1	Brain MRI: contrast‐enhanced T1WI‐MRI: Mild‐hyperintense asymmetrical involvement of bilateral cerebellar hemispheres and adjacent meninges with uniform contrast enhancement suggestive of infective cerebellitis	Not reported	Serum and cerebrospinal fluid results were negative
Ramkumarsingh Tomar et al. [46]	1	Brain MRI showed no significant findings	Not reported	Not reported
Cho et al. [48]	1	Brain MRI findings were not reported, while spinal MRI showed no significant findings	Not reported (nerve conduction velocity mentioned)	Serum findings were not reported, while cerebrospinal fluid was negative for anti‐myelin oligodendrocyte glycoprotein and aquaporin‐4 autoantibodies
Dalamapati et al. [49]	1	Brain MRI showed no significant findings	Not reported	Cerebrospinal fluid findings were not reported, while serum results were negative
Damodar et al. [50]	8	Brain MRI showed no significant findings	Not reported	Not reported
Nenjnktpaln [51]	1	Brain MRI showed no significant findings	Electroencephalogram demonstrated non‐specific slowing of wave activity	Cerebrospinal fluid findings were negative
Biswas et al. [52]	1	T2‐weighted, FLAIR, and DWI brain MRI revealed bilateral symmetrical hyperintense lesions in the basal ganglia, left parieto‐temporal cortex, and bilateral basifrontal areas	Electroencephalogram demonstrated generalised polyspike‐and‐wave discharges occurring at a frequency of 0.5–1 Hz	Both serum and cerebrospinal fluid findings were negative
Singh et al. [53]	1	Brain MRI demonstrated leptomeningeal enhancement in the cerebral hemispheres on post‐contrast imaging	Not reported	Not reported
Rehani et al. [54]	1	Not reported	Not reported	Not reported
Puppala et al. [55]	1	Brain MRI showed no significant findings	Not reported	Not reported
Yarlagadda et al. [56]	1	Brain MRI showed no significant findings	Not reported	Not reported
Dave et al. [57]	1	T2‐weighted and FLAIR brain MRI revealed hyperintensity in the bilateral cerebellum without evidence of diffusion restriction	Not reported	Not reported

Abbreviations: CSF, cerebrospinal fluid; DWI, diffusion‐weighted Imaging; FLAIR, fluid‐attenuated inversion recovery; MRI, magnetic resonance imaging.

Spinal cord imaging was reported in only 4 cases, with one case demonstrating features of acute transverse myelitis with intramedullary T2 hyperintensity and cord enlargement. Computed tomography imaging was performed in 6 cases, with most showing normal findings except for one case with a parietal calcified granuloma (Table 2).

Electrophysiological studies were reported in only 5 cases. One case showed generalised slowing on the electroencephalogram, while another demonstrated polyspike‐and‐wave discharges (Table 2). Autoimmune panels were performed in 12 cases, all of which were negative for various autoantibodies tested in both serum and CSF (Table 2).

Treatment details were available for 78.9% (60/76) of cases. Doxycycline monotherapy was the most frequently employed treatment strategy (36.7%, 22/60), while combined antibiotic therapy—most commonly with azithromycin—was also frequently used. Azithromycin monotherapy was used in 3.3% (2/60) (Table 1).

Outcomes were reported in 76.3% (58/76) of cases, with favourable results observed in most patients. Complete recovery was achieved in 81.0% (47/58), while partial recovery was noted in 8.6% (5/58), and residual deficits persisted in 10.3% (6/58) of cases. Among cases with documented recovery time, the median duration of recovery was 21 days, ranging from 7 to 90 days (Table 1).

Given the predominance of case reports and small case series and the lack of standardised quantitative outcome measures or control groups, a formal meta‐analysis was not feasible. Instead, we applied principal component analysis and correlation modelling to explore syndromic patterns and relationships among neurological features.

### Correlation analysis

**FIGURE 2:**
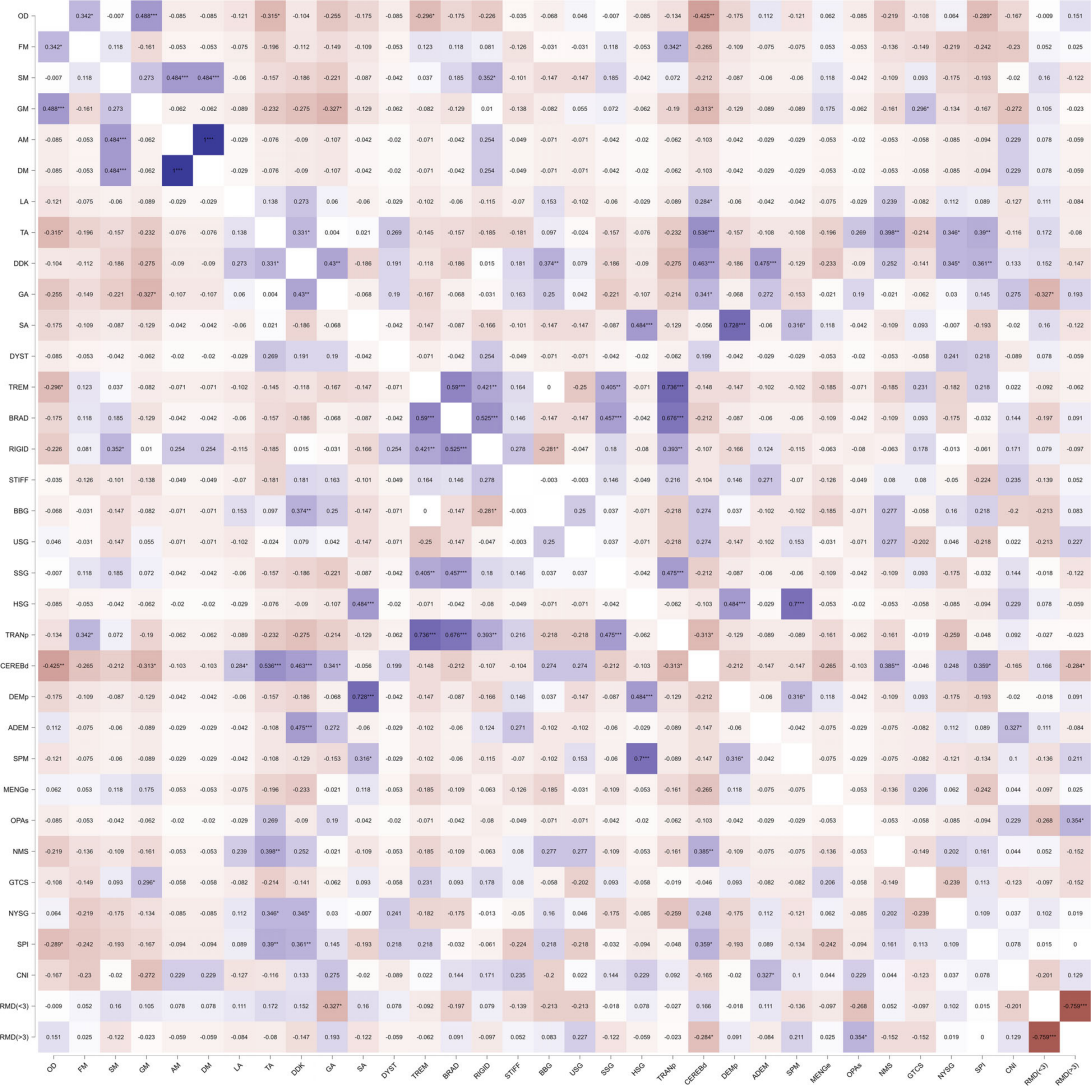
Correlation matrix displaying pairwise relationships among neurological features and movement or gait variables in patients with scrub typhus. Pearson's correlation coefficients (*r*) are colour‐coded: Blue indicates positive correlations, and red indicates negative correlations. More saturated colours represent stronger associations. Values are rounded to two decimal places. Statistically significant correlations (*p* < 0.05) are marked with an asterisk (*). Variables have been reordered to enhance clustering patterns. See Table 3 for a full list of abbreviations.

A correlation analysis was conducted to evaluate the relationships among neurological manifestations, recovery duration for movement and gait disorders, and the specific patterns of these disorders. The results, visualised in a heat map correlation plot (see Figure 2), present the correlation coefficients (*r* values) for each parameter. For clarity, shorthand variable names were used in the analysis; their full descriptions are provided in Table 3.

**TABLE 3:** Abbreviated neurological variables and their full descriptions for statistical and visual analyses.

Name	Meaning
OD	Oculomotor dyskinesia
FM	Focal myoclonus
SM	Segmental myoclonus
GM	Generalised myoclonus
AM	Action myoclonus
DM	Diaphragmatic myoclonus
LA	Limbic ataxia
TA	Truncal ataxia
DDK	Dysdiadochokinesia
GA	Gait ataxia
SA	Sensory ataxia
DYST	Dystonia
TREM	Tremor
BRAD	Bradykinesia
RIGID	Rigidity
STIFF	Stiffness
BBG	Broad‐based gait
USG	Unsteady gait
HSG	High stepping gait
TRANp	Transient Parkinsonism
CEREBd	Cerebellar dysfunction
DEMp	Demyelinating polyneuropathy
ADEM	Acute demyelinating encephalomyelitis
SPM	Myelopathy/myelitis
MENGe	Meningoencephalitis
OPAs	Opalski syndrome
NMS	Neuroleptic malignant syndrome
GTCS	Generalised tonic–clonic seizure
NYST	Nystagmus
SPI	Speech impairment
CNI	Cranial nerve involvement
RMD (<3)	Recovery of movement or gait disorder in less than 3 months
RMD (>3)	Recovery of movement or gait disorder after 3 months

The analysis revealed significant positive correlations between scrub typhus‐induced transient parkinsonism and several movement and gait disorders, including tremor (*r* = 0.736, *p* < 0.001), bradykinesia (*r* = 0.676, p < 0.001), short‐stepping gait (*r* = 0.475, *p* < 0.001), rigidity (*r* = 0.393, *p* < 0.01), and focal myoclonus (*r* = 0.342, *p* < 0.05). Conversely, transient parkinsonism showed a significant negative correlation with cerebellar dysfunction (*r* = −0.313, *p* < 0.05).

Cerebellar dysfunction was negatively correlated with oculomotor dyskinesia (*r* = −0.425, *p* < 0.01), generalised myoclonus (*r* = −0.313, *p* < 0.05), and prolonged recovery of movement or gait disorder (>3 months) (*r* = −0.284, *p* < 0.05). Positive correlations with cerebellar dysfunction included truncal ataxia (*r* = 0.536, *p* < 0.001), dysdiadochokinesia (*r* = 0.463, *p* < 0.001), neuroleptic malignant syndrome (*r* = 0.385, *p* < 0.01), speech impairment (*r* = 0.359, *p* < 0.05), and generalised ataxia (*r* = 0.341, *p* < 0.05).

Scrub typhus‐associated oculomotor dyskinesia showed positive correlations with generalised myoclonus (*r* = 0.488, *p* < 0.001) and focal myoclonus (*r* = 0.342, *p* < 0.05) but was negatively correlated with truncal ataxia (*r* = −0.315, *p* < 0.05) and tremor (*r* = −0.296, *p* < 0.05). Demyelinating polyneuropathy showed positive correlations with sensory ataxia (*r* = 0.728, *p* < 0.001), high‐stepping gait (*r* = 0.484, *p* < 0.001), and myelitis (*r* = 0.316, *p* < 0.05).

Acute demyelinating encephalomyelitis was positively correlated with dysdiadochokinesia (*r* = 0.475, *p* < 0.001) and cranial nerve involvement (*r* = 0.327, *p* < 0.05). In contrast, myelitis was correlated with high‐stepping gait (*r* = 0.700, *p* < 0.001) and sensory ataxia (*r* = 0.316, *p* < 0.05).

Additional findings included positive correlations between Opalski syndrome and a prolonged recovery period (>3 months) (*r* = 0.354, *p* < 0.05), neuroleptic malignant syndrome and truncal ataxia (*r* = 0.398, *p* < 0.01), generalised tonic–clonic seizures and generalised myoclonus (*r* = 0.296, *p* < 0.05), and nystagmus with truncal ataxia (*r* = 0.346, *p* < 0.05) and dysdiadochokinesia (*r* = 0.345, *p* < 0.05). Speech impairment correlated positively with truncal ataxia (*r* = 0.390, *p* < 0.01) and dysdiadochokinesia (*r* = 0.362, *p* < 0.01) and negatively with oculomotor dyskinesia (*r* = −0.289, *p* < 0.05). Recovery within less than 3 months was negatively correlated with generalised ataxia (*r* = −0.327, *p* < 0.05).

### Principal component analyses

**FIGURE 3:**
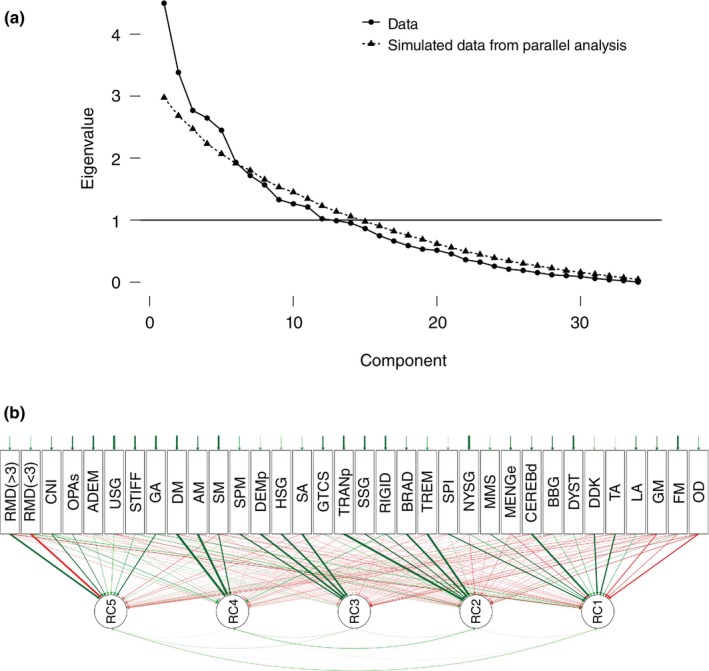
Principal component analysis of neurological features in scrub typhus‐associated movement and gait disorders. (a) A scree plot displays eigenvalues for each principal component. The *X*‐axis represents sequential components; the *Y*‐axis shows the eigenvalues indicating the variance explained. The dotted line represents observed eigenvalues; the triangle line denotes simulated eigenvalues from parallel analysis. The horizontal line at eigenvalue = 1 reflects the Kaiser criterion, used to retain components above this threshold. (b) Path diagram illustrating variable loadings onto the five retained components. Circles represent components; rectangles denote loading variables. Arrows indicate loading direction and strength, with green for positive and red for negative loadings. Arrow thickness corresponds to loading magnitude. See Table 3 for a full list of variable definitions.

Based on a selected eigenvalue of 1, determined from scree plot generation (see Figure 3a), five principal components (RC1–RC5) were identified (Table 4). The path diagram illustrates the components and their loading variables (see Figure 3b); full definitions of variable abbreviations are provided in Table 3.

**TABLE 4:** Principal component‐derived syndromic clusters of scrub typhus‐associated neurological manifestations.

Principal component analysis components	Top loading variables	Interpretation
RC1—Cerebellar dysfunction	Cerebellar dysfunction (0.812), dysdiadochokinesia (0.683), truncal ataxia (0.673), speech impairment (0.602), neuroleptic malignant syndrome (0.494); inverse: oculomotor dyskinesia (−0.593), generalised myoclonus (−0.502)	Cerebellar dysfunction as a central clinical feature; inverse relationship with brainstem‐related movements
RC2—Tremor‐parkinsonism	Tremor (0.899), transient parkinsonism (0.895), bradykinesia (0.818), short‐stepping gait (0.588), rigidity (0.537)	Tremor and parkinsonian features cluster together, suggesting basal ganglia involvement
RC3—Sensory ataxia and spinal involvement	Sensory ataxia (0.828), demyelinating polyneuropathy (0.804), high‐stepping gait (0.789), myelopathy (0.655)	Peripheral and spinal features cluster around sensory ataxia
RC4—Myoclonus (diaphragmatic/action/segmental)	Diaphragmatic myoclonus (0.950), action myoclonus (0.950), segmental myoclonus (0.615)	Cluster of myoclonic syndromes, primarily brainstem‐origin
RC5—Prolonged recovery and cranial nerve involvement	Prolonged recovery >3 months (0.781), gait ataxia (0.559), cranial nerve impairment (0.545), Opalski syndrome (0.433); inverse: recovery <3 months (−0.814)	Combination of prolonged recovery and brainstem/cerebellar deficits

*Note*: Values in parentheses indicate *factor loadings*, representing the strength and direction of association between each variable and the corresponding principal component. Loadings closer to +1 or−1 reflect stronger contributions, with negative values indicating inverse relationships.

RC1 primarily measured cerebellar dysfunction, strongly correlating with seven original variables. The component showed positive loadings with cerebellar dysfunction (0.812), dysdiadochokinesia (0.683), truncal ataxia (0.673), speech impairment (0.602), and neuroleptic malignant syndrome (0.494). These criteria tended to increase together. Conversely, RC1 exhibited negative loadings with oculomotor dyskinesia (−0.593) and generalised myoclonus (−0.502), indicating an inverse relationship with the other five variables. The highest loading, cerebellar dysfunction (0.812), suggested that more severe cases of scrub typhus were associated with cerebellar‐related pathologies.

RC2 was associated with tremor‐related symptoms and parkinsonism, with positive loadings for tremor (0.899), transient parkinsonism (0.895), bradykinesia (0.818), short‐stepping gait (0.588), and rigidity (0.537), with tremor showing the highest loading.

RC3 highlighted sensory ataxia, demyelinating polyneuropathy, and spinal involvement, with positive loadings for sensory ataxia (0.828), demyelinating polyneuropathy (0.804), high‐stepping gait (0.789), and myelopathy (0.655), sensory ataxia having the highest loading.

RC4 involved myoclonus‐related variables, showing positive loadings for diaphragmatic myoclonus (0.950), action myoclonus (0.950), and segmental myoclonus (0.615), with diaphragmatic and action myoclonus having the highest loadings.

RC5 related to recovery and cranial nerve impairment. This component exhibited positive loadings for a recovery period exceeding 3 months (0.781), gait ataxia (0.559), cranial nerve impairment (0.545), and Opalski syndrome (0.433). It had a negative loading for a recovery period <3 months (−0.814), indicating an inverse relationship with prolonged recovery. Among these, the recovery period >3 months had the highest loading.

In summary, the highest loadings across the five components (cerebellar dysfunction, tremor, sensory ataxia, diaphragmatic and action myoclonus, and extended recovery period) potentially represent key clinical features relevant to the diagnosis of scrub typhus‐induced movement and gait disorders (Table 4).

Interestingly, 10 variables—including focal myoclonus, limbic ataxia, dystonia, stiffness, broad‐based gait, unsteady gait, acute demyelinating encephalomyelitis, meningoencephalitis, generalised tonic–clonic seizure, and nystagmus—did not load onto any of the components, suggesting they may be less critical in identifying movement or gait disorders associated with scrub typhus.

### In‐silico findings of cross‐antigenic reactivity and neuro‐autoimmunity

A systematic literature review identified three major surface proteins associated with *Orientia tsutsugamushi* infection that serve as conserved epitopes across multiple serotypes: the 56 kDa type‐specific antigen (TSA56), *Orientia tsutsugamushi* deubiquitylase (OtDUB), and high‐temperature requirement A (HtrA, 47 kDa). TSA56 interacts with host fibronectin 1 (FN1), OtDUB with GOLGA1 and GOLGA2, and HtrA with human HTRA1.

**FIGURE 4:**
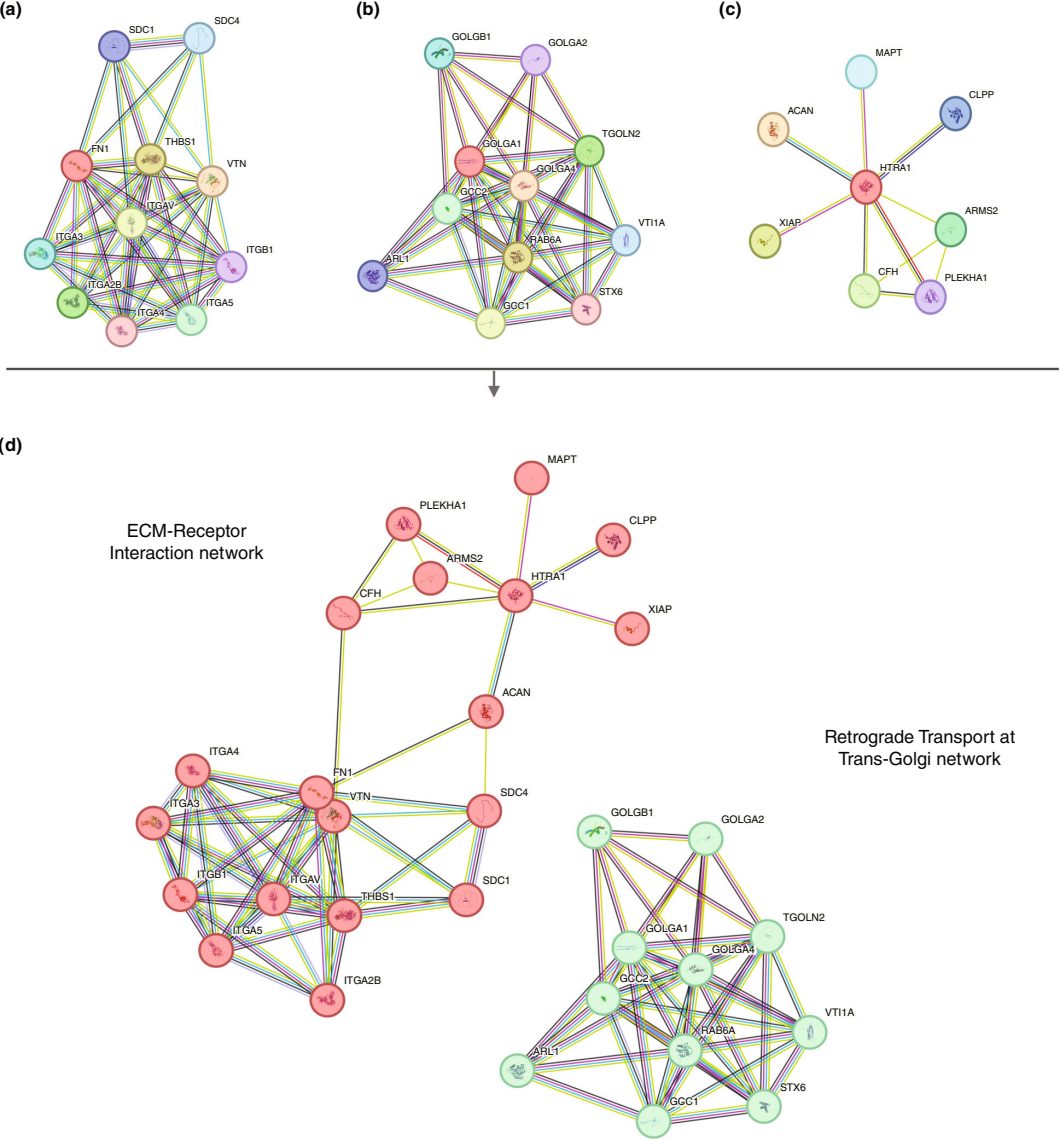
Host protein–protein interaction networks involving human proteins predicted to interact with *Orientia tsutsugamushi* antigens. Panels (A)–(C) illustrate the STRING‐based interaction networks of selected human proteins identified as likely targets of molecular mimicry by *Orientia tsutsugamushi* surface antigens. Panels (A) and (B) display host protein interaction networks for fibronectin‐1 (FN1) and for GOLGA1/GOLGA2, which are predicted targets of the bacterial proteins TSA56 and OtDUB, respectively. These Orientia proteins do not appear as nodes in the networks, as they are not part of the human STRING interactome; instead, their predicted human targets (FN1, GOLGA1, GOLGA2) are used as entry points to visualise the host‐side molecular context. Panel (C) shows the interaction network for HTRA1, a human protein functionally and structurally similar to the Orientia HtrA surface protein, highlighting its endogenous interactions. Panel (D) combines the networks into a comprehensive map of 30 human proteins, revealing two functionally distinct clusters: One centred on extracellular matrix (ECM) receptor interactions and another associated with retrograde transport in the trans‐Golgi network. These clusters reflect plausible biological pathways disrupted by host‐pathogen molecular mimicry. Nodes represent individual human proteins; coloured nodes mark query proteins. Edges indicate functional or physical protein–protein associations, with interaction evidence encoded by edge colour: Blue: Curated database. Pink: Experimental evidence. Green/Red/Dark Blue: Predicted (gene neighbourhood, fusions, co‐occurrence). Yellow/Light Blue: Text‐mining. Black: Co‐expression. These networks support the hypothesis that Orientia proteins disrupt host systems through mimicry of critical molecular pathways, such as ECM integrity and intracellular trafficking, potentially contributing to scrubtyphus‐associated neurological dysfunction.

Using STRING‐based PPI analysis, we identified 11 host proteins interacting with TSA56 and OtDUB and eight host proteins associated with HtrA (see Figure 4). A summary PPI network with a significant enrichment (PPI enrichment *p*‐value <1.0e‐16) was generated based on a total of 30 target host proteins. K‐means clustering revealed two primary clusters: one representing extracellular matrix (ECM)‐receptor interactions with 19 host proteins and another representing retrograde transport within the Trans‐Golgi Network (see Figure 4).

**FIGURE 5:**
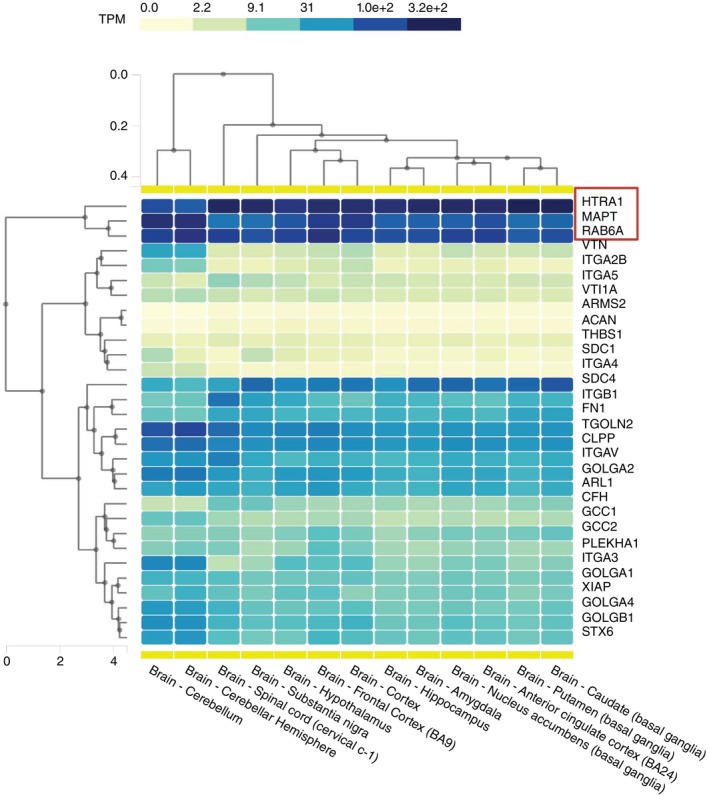
Tissue‐specific expression profiles of genes encoding the predicted host targets of Orientia tsutsugamushi, based on data from the genotype‐tissue expression (GTEx) Portal. Among the 30 host proteins analysed, HTRA1, MAPT, and RAB6A exhibited the highest expression in cerebellar and basal ganglia regions (highlighted in red). Expression levels are represented by a blue gradient, where darker shades indicate higher transcriptional activity. Values are reported in transcripts per million (TPM), providing a standardised measure across tissues. This figure underscores the regional specificity of gene expression in brain areas implicated in motor coordination, neuroinflammation, and infection‐related neurological syndromes.

Further analysis of tissue‐specific expression across different brain regions showed significant expression of these host proteins in the cerebellum and basal ganglia, highlighting their potential roles in these critical brain areas (see Figure 5). Among the 30 target proteins analysed, HTRA1, MAPT, and RAB6A displayed the highest levels of expression across all brain regions, indicating their importance in neural functions. In contrast, a subset of proteins, including SDC4, ITGB1, FN1, TGOLN2, CLPP, ITGAV, GOLGA2, ARL1, CFH, GCC1, GCC2, PLEKHA1, ITGA3, GOLGA1, XIAP, GOLGA4, GOLGB1, and STX6, exhibited moderate expression levels.

Finally, a group of proteins—VTN, ITGA2B, ITGA5, VTI1A, ARMS2, ACAN, THBS1, SDC1, and ITGA4—showed lower expression levels across the brain regions examined. These differential expression patterns may offer valuable insights into the specific roles and interactions of host proteins within the neural landscape.

## DISCUSSION

This review identified a broad spectrum of neurological manifestations. Among the 76 cases included, 50 exhibited isolated or combined movement disorders, most frequently opsoclonus, with the opsoclonus–myoclonus combination being the predominant presentation. Other hyperkinetic features included tremor and distinct myoclonus subtypes. A considerable proportion of cases exhibited parkinsonism. Gait abnormalities, excluding parkinsonian gait and instability due to myoclonus, were reported in 27 patients, with ataxic gait being the most frequent, predominantly of cerebellar origin.

Scrub typhus, though primarily an infectious disease, carries important neurological consequences that can result in significant morbidity and mortality. The infection‐induced inflammatory cascade can trigger a range of central nervous system manifestations, underscoring the need for early recognition and prompt treatment [7, 58, 59].

Principal component analysis enabled the identification of five clinically coherent symptom clusters: cerebellar dysfunction, tremor and parkinsonism, sensory ataxia and spinal involvement, myoclonus (diaphragmatic/action/segmental), and prolonged recovery and cranial nerve involvement. Each component likely reflects a distinct pathophysiological axis—ranging from neuroimmune disruption of the cerebellum to basal ganglia inflammation and peripheral demyelination.

Unlike traditional clustering techniques, which assign cases to mutually exclusive diagnostic groups, principal component analysis offers a dimensional, syndromic framework that reflects how features overlap in real‐world clinical presentations. In the context of scrub typhus—a disease with pleomorphic neurological features—this approach is particularly valuable. It allows for a data‐driven examination of how neurological symptoms co‐occur within individual cases and across the cohort, uncovering underlying patterns that may reflect shared pathophysiological mechanisms or point towards potential therapeutic targets. To minimise the risk of misclassification, we included only cases with microbiological confirmation.

Our in‐silico modelling supports the hypothesis of molecular mimicry and cross‐antigenic reactivity. STRING‐predicted PPI networks identified targeted host proteins—including FN1, GOLGA1, GOLGA2, and HTRA1—interacting with *Orientia tsutsugamushi* antigens (TSA56, OtDUB, and HtrA). While not yet experimentally validated, these predicted interactions suggest plausible disruptions to ECM integrity and retrograde Golgi trafficking, both implicated in central nervous system dysfunction. Several implicated host proteins—notably HTRA1, MAPT, and RAB6A—are highly expressed in the cerebellum and basal ganglia, aligning with observed clinical features such as ataxia, opsoclonus‐myoclonus, and parkinsonism [60]. These findings suggest HTRA1, MAPT, and FN1 as potential biomarkers of immune‐mediated neurological injury in scrub typhus.

Neuroimaging provided further insight, with T2‐weighted hyperintensities in the cerebellum, basal ganglia, and leptomeninges—findings consistent with both inflammatory and ischaemic injury. These patterns may aid in early recognition of scrub typhus–associated central nervous system involvement.

Despite these advances, the study has limitations. Heterogeneity in reporting standards, variable sample sizes, and the inclusion of case reports and case series restrict generalisability and preclude risk estimation. Language bias may also be present due to the inclusion of English‐only studies. Additionally, the lack of standardised assessment tools for infectious movement disorders complicates cross‐study comparisons.

Future research should aim to clarify causal links between *Orientia tsutsugamushi* infection and neurological outcomes. Mechanistic investigations into host immune responses and longitudinal studies tracking recovery trajectories are needed. The syndromic structure delineated by principal component analysis may provide a foundation for future clinical phenotyping, biomarker validation, and the development of targeted immunotherapies.

In conclusion, scrub typhus is an underrecognized yet clinically significant cause of movement and gait disorders, likely mediated by neuroimmune mechanisms and molecular mimicry. Using principal component analysis, we identified syndromic clusters that offer a data‐driven framework for classifying these neurological manifestations and advancing diagnostic precision. Our findings emphasise the importance of early recognition and timely, targeted treatment to improve neurological outcomes. In endemic regions, prompt initiation of doxycycline remains essential to reducing the risk of long‐term complications. Future research should focus on validating the host‐pathogen interactions identified through in‐silico modelling and exploring immunomodulatory therapies as potential avenues for intervention.

## FUNDING INFORMATION

No specific funding was received for this work.

## CONFLICT OF INTEREST STATEMENT

The authors declare that there are no conflicts of interest relevant to this work.
